# MiRNAs at the Crossroads between Innate Immunity and Cancer: Focus on Macrophages

**DOI:** 10.3390/cells7020012

**Published:** 2018-02-08

**Authors:** Graziella Curtale

**Affiliations:** Department of Immunology and Microbiology, the Scripps Research Institute, Jupiter, 33458 FL, USA; gcurtale@gmail.com; Tel./Fax: +1-561-228-2372

**Keywords:** microRNA, inflammation, cancer, macrophages

## Abstract

Innate immune cells form an integrative component of the tumor microenvironment (TME), which can control or prevent tumor initiation and progression, due to the simultaneous processing of both anti- and pro-growth signals. This decision-making process is a consequence of gene expression changes, which are in part dependent on post-transcriptional regulatory mechanisms. In this context, microRNAs have been shown to regulate both recruitment and activation of specific tumor-associated immune cells in the TME. This review aims to describe the most important microRNAs that target cancer-related innate immune pathways. The role of exosomal microRNAs in tumor progression and microRNA-based therapeutic strategies are also discussed.

## 1. Introduction

In the last decade, local immune response has emerged as key factor in the modulation of the multistep process of cancer progression (reviewed in [1,2]). Innate and adaptive immune cells not only infiltrate tumors themselves, but also the tumor milieu. The highly heterogeneous tumor microenvironment (TME) consists of fibroblasts, immune cells, endothelial cell progenitors, extracellular matrix components, blood, and lymphatic vessels (reviewed in [3,4]). Altogether, these different cell types contribute to tumor initiation and progression, by regulating a fine balance between anti- and pro-growth signals. Indeed, the immune system and tumor cells constantly engage each other in a dynamic process that can have synergistic or opposite outcomes. These include eradication of the tumor, according to the “tumor surveillance hypothesis”, coined by Burnet and Thomas in 1957 [5,6,7], or the escape of tumor from immune recognition [1,8]. Evidence supporting the involvement of the immune system in the recognition and eradication of developing tumors was provided by in vivo experiments on immunodeficient mice, including Rag2^−/−^, IFNγ^−/−^ or Prf^−/−^ genetic backgrounds [9,10,11,12,13]. These studies showed higher incidence of spontaneous and carcinogen-induced solid tumors as well as an increased incidence of lymphomas [13]. However, cancer cells can also generate their own immunosuppressive microenvironment that can be traced to the transcriptional regulatory level [14]. 

In this review we will focus on the role of miRNAs as molecular determinants in tumor progression and macrophage-mediated immune surveillance.

### 1.1. MiRNA Biogenesis and Maturation: A Brief Overview

MicroRNAs (miRNAs) are small noncoding RNAs endogenously expressed in almost all eukaryotes, and are considered major epigenetic factors in post-transcriptional regulation of gene expression [15,16,17,18]. These 20–2 nucleotide (nt) molecules are mainly transcribed by RNA polymerase II as primary miRNA transcripts, termed pri-miRNAs [19,20]. They are then subjected to an extensive processing in the nucleus as substrates of a large protein complex called the microprocessor, that includes the RNAse III enzyme Drosha and its partner DiGeorge syndrome critical region 8 (DGCR8) [16,21,22,23]. This first maturation step transforms pri-miRNA into stem-loop-structured miRNA precursors (pre-miRNAs), which are then exported to the cytoplasm through the Exportin 5 (Xpo5) system [21,24,25]. There, another RNAse III protein, called Dicer, cuts pre-miRNA molecules into mature miRNAs [16,21,26]. Suitably processed, mature miRNA molecules are finally incorporated into the RNA-induced silencing complex (RISC) [27,28]. The core component of RISC is a member of the Ago protein family (most commonly Ago2), which brings together the templating miRNA and its target mRNA [16,29]. miRNA-loaded RISC specifically recognizes short sequences of 6–7 nt, located in the 3′ untranslated region (UTR) of target mRNAs, that are complementary to the so-called “seed region”, located in the 5′ end of the miRNA molecule [30,31,32]. MiRNAs can either induce mRNA degradation or inhibition of translation, depending on the degree of complementarity between the 3′UTR and the seed region [31,33,34]. 

### 1.2. Dysregulation of miRNA Expression and Biogenesis in Cancer

It has been estimated that approximately 30–60% of human protein-coding genes are regulated by miRNAs [35,36,37,38,39,40]. MiRNAs participate in modulating several biological processes, including differentiation, apoptosis, cell cycle, proliferation, and the immune response [41,42]. Alterations in miRNA expression profile have been associated with several diseases, including cancer [43,44,45,46]. 

Deregulated miRNA expression favors acquisition of cancer hallmarks as well as affects the tumor microenvironment, leading to development and progression of tumor. 

**Figure 1:**
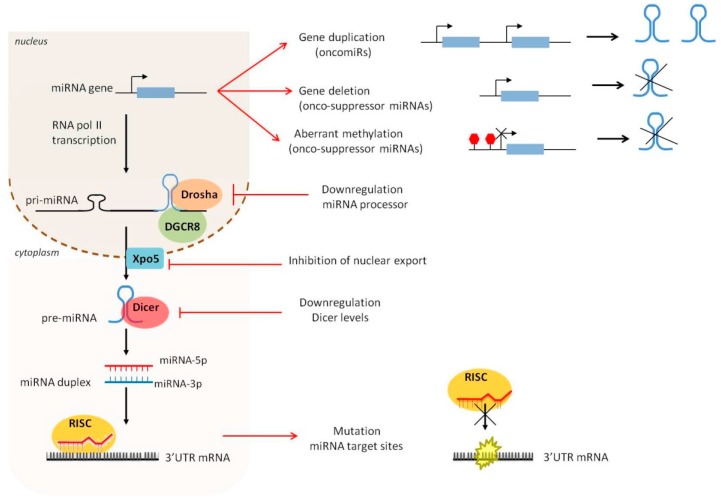
Deregulation of microRNA (miRNA) biogenesis during cancer initiation and progression.

Both genetic and epigenetic mechanisms have been involved in miRNA expression deregulation in cancer; including amplification or deletion of miRNA genes, acetylation and methylation of miRNA promoter, aberrant transcriptional control and defects in the miRNA biogenesis machinery [47,48,49] (Figure 1). 

During cancer initiation and progression, deregulation of miRNA expression can occur at different steps of miRNA biogenesis. Gene duplication of the miRNA promoter can lead to enhanced transcription of the pri-miRNA molecule. Aberrant methylation of the miRNA promoter, and reduced expression of factors involved in miRNA processing and mutation of the seed site in the 3′UTR of mRNA targets can abrogate miRNA maturation or functional activity.

#### 1.2.1. Amplification and Deletion of miRNA Genes

More than 50% of miRNA genes are located in fragile sites or cancer-associated genomic regions [47,50]. The first study of altered miRNA expression in cancer showed that miR-15 and miR-16, targeting the anti-apoptotic factor B cell lymphoma 2 (BCL2), are located at a chromosomal region (13q14), frequently deleted in the majority of chronic lymphocytic leukemia (CLL) cases [50]. Since then, other studies in both human and mice cells demonstrated the association between genomic location of miRNA and cancer susceptibility [50,51]. In 2010, Lagana et al. investigated the location of more than 700 miRNA loci at chromosomal fragile sites and found that 44% of them were located in genomic regions frequently translocated in cancer. The most frequent deleted miRNAs were the miR-204 and the let-7 family, both reported as onco-suppressor miRNAs [52,53,54].

#### 1.2.2. Epigenetic Alterations

miRNA loci are frequently amplified or deleted in concert with epigenetic alterations. In some cases they show aberrant epigenetic marks, such as DNA hypo/hypermethylation and hypo/hyperacetylation at the promoter of miRNAs, or repressive histone marks, that lead to the silencing of the miRNA gene [55,56,57,58,59] (Figure 1). Genome-wide screening studies in cancer cells identified specific epigenetic patterns associated with active or repressed miRNA promoter. In particular, tri-methylation of histone H3 lysine 4 (H3K4me3) and acetylation of histone H3 lysine 9 or 14 (H3K9ac and H3K14ac, respectively) are hallmarks of active miRNA gene promoters. By contrast, H3K9me2, H3K9me3 and H3K27me3 are marks of repression [33,43]. The characterization of specific epigenetic marks on miRNA promoters enabled the identification of epigenetically dysregulated miRNAs in both solid and hematological malignancies [60,61].

Aberrant DNA methylation is another frequent epigenetic mechanism of miRNA gene silencing. In normal cells, the CpG island is unmethylated and the chromatin is associated with active histone modifications (H3K4me3, H3K3me2, H3K9ac and H3K14ac). In cancer cells, miRNAs that exert a tumor suppressor function are downregulated via CpG island hypermethylation of the corresponding gene, while miRNAs with a pro-tumoral activity are often upregulated via hypomethylation of their CpG island. For instance, hypermethylation of CpG island at the promoter of members of the miR-34 gene family (miR-34a, miR-34b, and miR-34c), frequently occurs in multiple cancer types, [55,61] and the aberrant methylation of miR-34 has also been associated with increased frequency of cancer metastasis and invasion. [16,31]. Another miRNA epigenetically silenced in both solid and hematological malignancies is the miR-124 family (miR-124-1, miR-124-2, and miR-124-3), a tumor suppressor miRNA, which targets RAC1 and cyclin-dependent kinase 6 (CDK6) [47,48,62,63]. Its reduced expression in all patients results in the activation of CDK6 and the phosphorylation of retinoblastoma (Rb) protein [44]. 

#### 1.2.3. Defects in the miRNA Biogenesis Pathway

Downregulation of enzymes and cofactors involved in the miRNA maturation process can also lead to dysregulated miRNA expression in cancer cells. Evidence reported in the last decade demonstrates that many proteins involved in miRNA processing function as haplo-insufficient tumor suppressors. For instance, mutations of Xpo5 that trap miRNA transcripts in the nucleus correlate with carcinomas characterized by microsatellite instability [64,65]. Similarly, partial deletions of Dicer1 and Drosha have been shown to promote tumorigenesis in vitro and in vivo [62,66]. Moreover, recent findings demonstrated that two subunits of the microprocessor: the DEAD-box RNA helicase p68 (DDX5) and p72 (DDX17), required for the recruitment of the microprocessor to some pri-miRNAs, are also substrates of the tumor suppressor p53 [67]. In response to DNA damage, p53 interacts with p68 and enhances the post-transcriptional maturation of miRNAs with growth-suppressive function [67,68]. Conversely, mutant versions of p53 have been shown to inhibit post-transcriptionally the maturation of a subset of onco-suppressor miRNAs, by sequestering the RNA helicase p72/82 from the microprocessor complex [68]. These findings suggest that p53 governs a tumor suppressive program that affects miRNA biogenesis in a transcription-independent fashion.

#### 1.2.4. Mutations of miRNA Target Genes

A few studies also reported genetic alterations in miRNA target sites in the 3′UTR of onco-suppressor genes [69,70]. In particular, two different papers published in 2007 described the presence of chromosomal rearrangements at the 3′UTR of the HMGA2 locus, resulting in the escape of HMGA2 from let-7 regulation and the consequent promotion of tumor formation [70,71]. Additionally, a clinical study of 87 non-small cell lung cancer patients published by Chin et al. [72] identified a single nucleotide polymorphism (SNP) in the 3′UTR of the KRAS oncogene. This variant allele caused the disruption of let-7e-mediated regulation of KRAS and the consequent upregulation of KRAS expression correlating with increased cancer risk [72]. 

## 2. Role of miRNAs in Macrophage Polarization

Macrophages are key players at the interface between innate and adaptive immunity, acting as antigen-presenting cells, phagocytes and able to promote inflammation and also its resolution through differentiated immune lineages. One of the most remarkable characteristics of macrophages is their plasticity and heterogeneity. According to their responsiveness to specific environmental cues, they are generally classified into two main types: M1 (classically activated) and M2 (alternatively activated) macrophages, with each displaying a characteristic gene expression profile and functional phenotype. M1-polarized macrophages are potent antimicrobial effector cells, specialized in the defense against intracellular pathogens. They are responsive to pro-inflammatory stimuli like interferon-γ (IFNγ) and lipopolysaccharide (LPS). Both IFNγ- and LPS-mediated activation of TLR4 converge on the activation of: nuclear factor kappa-B (NF-κB), activator protein 1 (AP-1) and activator of transcription 1 (STAT1), leading to the release of pro-inflammatory cytokines (i.e., TNFα, IL-12, IL-1, IL-6) and chemokines (i.e., chemokine (C-C motif) ligand 2, CCL2, chemokine (C-X-C motif) ligand 10, CXCL10). By contrast, M2-polarized macrophages are specialized in defending against extracellular pathogens and are induced by a broader range of stimuli, including IL-4, IL-10, IL-13, and glucocorticoids. M2 macrophages are reviewed in [73] (Table 1). 

Several studies have profiled the expression of miRNAs in M1- and M2-polarized human and murine macrophages, by using microarray and quantitative polymerase chain reaction (qPCR) techniques. A report published in 2013 by Zhang et al. [74] showed the expression profile of murine bone marrow-derived macrophages (BMdM), identifying 109 miRNAs differentially expressed between M1- and M2-polarized conditions. More specifically, miR-155, miR-181 and miR-451 were significantly upregulated in M1 macrophages, whereas miR-125a-5p, miR-146a, miR-145-5p, miR-143-3p were more highly expressed in M2 macrophages. Later studies confirmed the upregulation of miR-155 in classically activated macrophages and the increased expression of miR-146a, miR-125b-5p and miR-127 in M2 macrophages [75]. Other miRNAs associated to M2 polarization were also identified; in particular miR-223 and let-7c [76].

miR-155 is one of the first miRNAs characterized as involved in both innate and adaptive immunity [77,78,79,80]. It plays a central role in the acquisition and modulation of M1/M2 profile, as it affects the expression of several genes important for the immune balance. miR-155 directly targets the expression of the IL-13 receptor α1 (IL13Rα1), thereby inhibiting STAT6 activation and promoting M1 polarization [81]. Genes related to M2/pro-Th2 phenotype in macrophages indirectly regulated by miR-155 include CCL18, SERPINE, CD23 and DC-SIGN [81].

Other miRNAs that promote M1 polarization are miR-125b and miR-127. Taganov et al. demonstrated that miR-125b overexpression enhanced responsiveness to IFNγ, through the targeting of IRF4 and increased expression of pro-inflammatory cytokines [82]. These alterations in cell signaling and gene expression also resulted in increased killing of EL4 tumor cells both in vitro and in vivo [82]. 

Promotion of the M1 phenotype by miR-127 is mechanistically related to the inhibition of Bcl6 expression, which inhibits the phosphatase Dusp1. Reduction of Dusp1 levels lead to increased phosphorylation of JNK and the consequent induction of the inflammatory response. Accordingly, knockdown of miR-127 suppressed the expression of M1 signature genes and promoted the transcription of M2-related genes [83].

One of the first miRNA showing anti-inflammatory properties associated with M2 polarization is miR-146a. Transfection of peritoneal macrophages with miR-146a reduced the expression of M1 phenotype markers (e.g., iNOs, CD86, TNFα, IL-12 and IL-6), and increased the production of M2 marker genes (e.g., Arg1, CCL17, CCL22 and CD206) [84]. Mechanistically, miR-146a promotes M2 polarization at least in part by targeting Notch1, PPARγ and inhibin βA subunit of activin A (INHBA) [84,85].

miR-223 and let-7c have been shown to initiate M2 polarization in murine RAW264.7 macrophages. Enforced expression of miR-223 lead to the inhibition of LPS-dependent release of IL-1β and IL-6 [86]. Similarly, overexpression of let-7c reduced the expression of M1-related genes (i.e., iNOS and IL-12) and increased levels of M2 markers (i.e., FR-β), via targeting of C/EBP-δ [87] and PAK1 [88].

## 3. The Fine Balance of the Innate Immune Tumor Response

While the importance of adaptive immunity in tumor surveillance is recognized, the role of innate immune cells in this context has been subject to extensive debate. Evidence demonstrates that innate immune response can be critical in tumor prevention, but also in its initiation and progression. The final outcome of pro- versus anti-tumoral innate immune response is dependent on the TME, which first primes and then reinforces the differentiation and response of leukocytes recruited or already present in situ. Tumor milieu composition is not static, but dynamically evolves with tumor progression to promote growth and metastatic potential of cancer cells. Increasing evidence indicates that the majority of solid tumors are strictly linked to a chronic inflammation that reciprocally affects the cellular and molecular composition of the TME. Chronic inflammation is induced by pro-inflammatory mediators, released by myeloid cells. During the first stages of tumor formation, pro-inflammatory macrophages (e.g., M1 macrophages) represent the predominant type of myeloid cells involved. M1 macrophages not only release pro-inflammatory mediators, but are also characterized by increased phagocytic ability [89]. However, chronic inflammation can create a mutagenic microenvironment, causing genomic instability in the cells present. Due to this genetic instability, cancer cells acquire features that render them resistant to pro-inflammatory immune response and able to evade tumor immunosurveillance, promoting M2 macrophages for that purpose [90,91]. An example of an immune escape mechanism exploited by cancer is the upregulation of CD47 expression on tumor cells of patients affected by myeloid leukemia. CD47 is a pentaspanin, ubiquitously expressed on mammalian tissues, also known as integrin-associated protein (IAP) [92]. The engagement of CD47 with signal regulatory protein alpha (SIRPα) expressed on macrophages, leads to inhibition and evasion of phagocytosis [93,94,95]. 

A second step in cancer progression is the infiltration of malignant tumors by pro-tumor macrophages: a heterogeneous population predominantly composed by tumor-associated macrophages (TAMs) and myeloid-derived suppressor cells (MDSCs). They both play a critical role in malignancy by promoting tumor growth, angiogenesis, and lymph node metastasis [96]. The dual role of macrophages in both preventing and promoting cancer progression reflects the complexity of the innate immune system in tumor development. Emerging evidence shows that this is apparent dualism is not due to recruitment of distinct differentiated cell types, but is rather from the result of extracellular stimuli as well as genetic and epigenetic changes that dynamically shape the innate immune cells [97]. Quality and duration of the innate immune response will ultimately dictate the fate of cancer development and the consequent clinical prognosis. In this context, the molecular mechanisms of crosstalk between cancer and immune cells are object of extensive studies and still remain to be extensively elucidated.

## 4. miRNAs Involved in Tumor Immunosurveillance 

Tumor immunosurveillance is a well-established mechanism for regulation of tumor growth. In this regard, most studies have focused on the role of T- and NK-cells as critical immune effector cells (reviewed in: [98,99,100]). However, TAM play a major role in the recognition and clearance of foreign and damaged cells. They can infiltrate solid tumors and modulate T cell activity within the TME [101,102,103,104]. The general consensus is that TAM are usually pro-tumorigenic [105]. They are recruited by tumor-derived chemokines and produce low levels of inflammatory cytokines, promote Th2-T cell response, favor wound healing, and increase angiogenesis and metastases [106,107,108]. 

MDSCs are among the major myeloid cells responsible for cancer immune evasion [109]. They represent a heterogenic population of immature myeloid cell progenitors, macrophage precursors, granulocytes and dendritic cells (reviewed in [110,111]). They can promote tumor growth by enhancing angiogenesis or suppressing innate and adaptive immune responses [112,113,114]. In particular, MDSCs suppress T-cell responses by secretion of immune modulatory factors [75,115], restriction of T cell homing [116], induction of Tregs [117], suppression of NK cell cytotoxicity [118,119], promotion of M2 macrophage differentiation [120], and modulation of the priming activity of mature dendritic cells. Although TAMs and MDSCs are often regarded as separate entities, they share many characteristics in terms of gene expression profile [121]. For instance, they both require CCL2/CCR2 signaling for their recruitment to tissue sites [122,123]. Extensive studies provided evidence relative to aberrant expansion and accumulation of MDSCs in tumor models, and the molecular mechanism involved has started to be elucidated [124,125,126].

Recently, miRNAs have been identified as important determinants of TAM pro-tumoral activity as well as for the accumulation, expansion and tumor promoting function of MDSCs. A paradigmatic example is represented by miR-155, which mediates the antitumor potential of myeloid cells in early stages of breast cancer carcinogenesis [87]. miR-155 knockdown in myeloid cells induces faster tumor growth, reduction of M1-macrophages and enrichment of pro-tumor cytokines within tumor milieu. The proposed molecular mechanism to explain the observed phenotype involves the regulation of SHIP1, a major negative regulator of the pro-inflammatory PI3K/AKT pathway. The inhibition of this pathway was demonstrated to revert the common pro-inflammatory and pro-tumor events mediated by AKT activation [77,127]. miR-155 is also fundamental in the recruitment of MDSCs to the tumor site. In particular, it has been recently demonstrated to mediate the recruitment of MDSCs in glioma [128]. Similarly, miR-494, whose expression is induced by tumor-derived factors, enhances CXCR4-mediated chemotaxis of MDSCs and also modulates the intrinsic apoptotic pathway by targeting phosphatase and tensin homolog (PTEN) [82]. This results in increased activity of the Akt1 pathway and upregulation of MMPs, involved in tumor invasion and metastasis [82,129]. Other miRNAs such as miR-223, miR-21, and miR-690, have been recently described as important factors that affect differentiation and functional activity of MDSCs [130,131]. miR-223 is largely expressed in myeloid cells and downregulated in tumor associated MDSCs. It was first characterized as negative modulators of granulocytes differentiation and activation [132], and then demonstrated to suppress differentiation of tumor-induced CD11b^+^Gr1^+^ MDSC [133]. miR-21 is upregulated in bone marrow-derived and splenic MDSCs. It regulates STAT3 activation by targeting phosphatase and tensin homolog (PTEN) protein [134]. miR-690 is upregulated in EL-4 tumor-elicited MDSCs. A study published in 2013 by Hedge et al. demonstrated that miR-690 maintains MDSCs at their immature immunosuppressive state by targeting CEBP/α, a transcription factor involved in cell cycle progression and terminal differentiation of myeloid cells [135].

## 5. TAM-Derived miRNAs in Cancer-Related Inflammation

Inflammation has been defined in 2009 as a hallmark of cancer [136]. Chronic and persistent inflammation can predispose cells to carcinogenesis and contribute to cancer development. Cancer-associated inflammation is characterized by the presence of infiltrating leukocytes, cytokines, chemokines, matrix-degrading enzymes and growth factors. This persistent inflammatory state can be initiated by microbial, viral or parasitic infection, and is responsible for the pathogenesis of about 15–20% of human tumors [137]. However, even tumors that are not epidemiologically linked to pathogens are characterized as having inflammatory infiltrates. Two general molecular pathways have been proposed to describe the interaction between inflammation and cancer: the intrinsic and the extrinsic pathways (reviewed in [131,137,138]). The intrinsic pathway consists of a series of genetic events (e.g., activation of oncogenes, inactivation of tumor suppressor genes) leading to neoplastic transformation that initiates inflammation-related programs. In the extrinsic pathway, tumor-infiltrating leukocytes, in particular macrophages, establish a state of chronic inflammation. Thus, both pathways converge in the activation of pro-inflammatory transcription factors in cancer cells and in the subsequent production of inflammatory mediators (i.e., cytokines, chemokines and prostaglandins) [139]. Evidence suggests that the inflammatory microenvironment so created further induces genetic instability in developing tumor cells, and promotes further infiltration by resident immune cells such as macrophages [139,140], MDSCs, [141], neutrophils [142], and mast cells [143,144]. In this context, TAMs represent a central linker between chronic inflammation and cancer [139,145]. Regardless of their origin, these immune signals present in the TME play a crucial role in all stages of cancer evolution, from initiation to metastasis [101,146,147]. The release of pro-inflammatory mediators represents a central step in the cross talk between immune and cancer cells. It is driven by the activation of key transcription factors in response to different upstream stimuli (e.g., infection and pro-inflammatory cytokines) [145]. Among them, NF-κB and signal transducer and activator of transcription 3 (STAT3) have been shown to modulate the activation of numerous oncogenic pathways [148,149,150,151,152,153]. 

**Figure 2:**
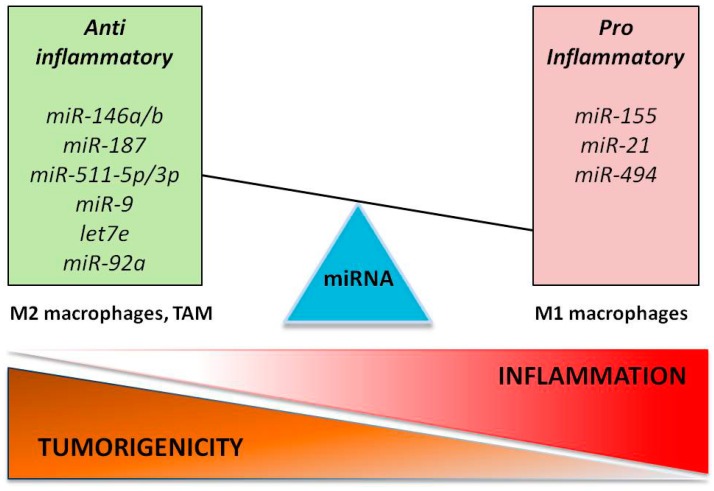
MiRNAs expression in macrophages modulates inflammation and carcinogenesis. MiRNAs modulate macrophage polarization from a pro-inflammatory M1 to an anti-inflammatory phenotype that inversely correlates with the tumorigenic potential of macrophages during carcinogenesis.

Notably, the regulation of these critical proteins and their relative pathways is extensively regulated at a post-transcriptional level by miRNAs (Figure 2). NF-κB is a major regulator of innate immunity and inflammation [150]. Downstream effects of NF-κB are cell-type dependent, consisting of induction of pro-inflammatory gene expression in immune cells, and of anti-apoptotic genes in tumor cells, favoring tumor development. A study published in 2009 by Bazzoni et al. [154] demonstrated the direct targeting of the NF-κB1 transcript by miR-9, with consequent reduction of pro-inflammatory cytokines. Due to the inflammatory microenvironment and oncogenic mutations, a significant number of human cancers have constitutive NF-κB activity that results in increased tumor proliferation and angiogenesis [155]. miR-9 deregulation in colon cancer cells has been consistently shown to suppress apoptosis and promote proliferation and tumor survival [156,157]. Other miRNAs have also been linked to the regulation of NF-κB signaling. In particular, miR-146a is involved in the control of inflammatory response. In particular, NF-κB-dependent miR-146a expression is induced in monocytes and macrophages upon triggering of TLR4 by LPS or other pro-inflammatory stimuli [128]. It downregulates the expression of the two adaptor proteins IRAK1 and TRAF6 (downstream of toll-like and cytokine receptors) and represses NF-κB signaling [128]. The miR-146a/NF-κB axis has been investigated in gastric and breast cancer cells, confirming the targeting of IRAK1 and TRAF6, and the consequent reduction of metastatic potential [133]. Two of the most important activators of NF-κB signaling are IL-6 and TNFα, which bind to their specific receptors (i.e., IL-6R and TNFR1/2, respectively) expressed by immune or cancer cells [158]. IL-6 and TNFα are both pro-inflammatory cytokines, exerting pro-tumoral functions, including the promotion of angiogenesis and metastasis [159,160,161,162]. They are downregulated in human macrophages by several anti-inflammatory miRNAs, such as miR-187 [163], miR-146a [128], let-7e [164], and miR-92a [165] as a functional consequence of the direct targeting of molecular components participating in TLR signaling (Figure 2). STAT3 is constitutively activated in both tumor and immune cells by IL-6. STAT3 signaling is required for immunosuppressive and pro-tumorigenic function of both innate (MDSCs, TAMs) [166] and adaptive (Treg and Th17 lymphocytes) immune cells [167]. One of the first miRNAs described as an important regulator of STAT3 expression is miR-21. Expressed in both immune and cancer cells, miR-21 displays pro-inflammatory and pro-tumoral activity, respectively. miR-17-5p and miR-20a are two other important modulators of STAT3 expression, downregulated in MDSCs by tumor associated factors [168]. Ectopic expression of either miR-20a or miR-17-5p decreases MDSCs’ ability to suppress antigen-specific CD4 and CD8 T cell responses [168]. Immunosuppressive and anti-inflammatory signals derive also from the actions of IL-10 and transforming growth factor β (TGF-β), which have a general role in tumor suppression by inhibiting the production of pro-inflammatory cytokines [169,170,171,172]. They activate a negative feedback loop mechanism that induces the expression of other anti-inflammatory miRNA, such as miR-146b and miR-511-5p/3p [101,145,162]. These miRNAs have been recently described as part of the complex immune regulatory network of macrophage-derived anti-inflammatory response. Their specific role in in vivo tumor models has not been explored yet. Further investigations are needed to fully elucidate how miR-146b and miR-511 contribute to the antitumor immune response. 

Altogether, this evidence strongly supports the notion that miRNAs participate in MDSCs and TAM-mediated modulation of the local inflammatory tumors. Our improved understanding of the connection between miRNAs, inflammation and cancer may provide novel preventive, diagnostic and therapeutic strategies.

## 6. Exosome-Derived miRNAs in Cancer Progression and Metastasis

**Figure 3:**
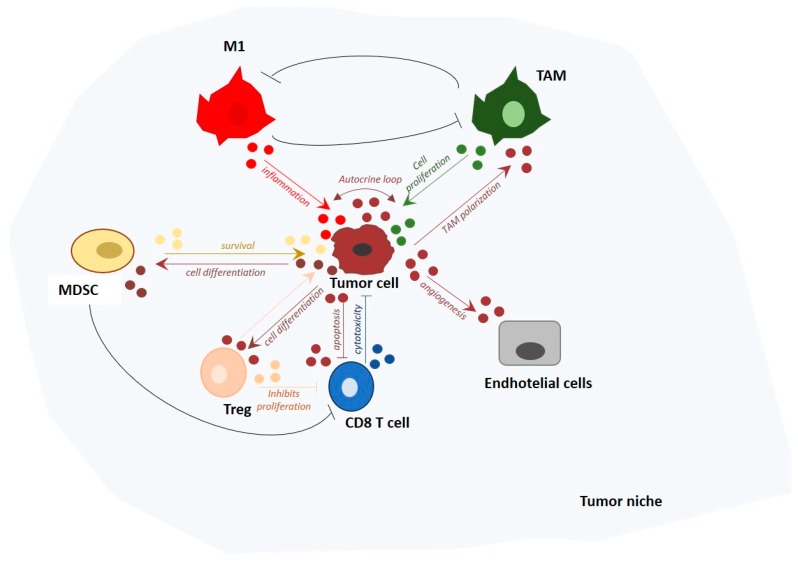
Exosomal miRNAs as molecular messages exchanged between immune cells and tumor. The tumor microenvironment (TME) is a highly heterogeneous environment where different cell types, including macrophages, T lymphocytes, and endothelial cells, interact with each other by releasing exosomal miRNAs. The result of this interaction can lead to cell differentiation, survival, apoptosis, or angiogenesis, and strongly affects tumor initiation and progression.

Apart from their unique role as cytoplasmic regulators, recent evidence suggests that miRNAs are also components of a complex mechanism of indirect cell-to cell communication. They act as intercellular messengers transferred from donor to recipient cells through extracellular vesicles such as exosomes, altering gene expression in neighboring and even distant cells. Exosome-derived miRNAs are thought to play an important role in cell-to-cell communication, particularly in the cross talk between immune and tumor cells within the TME [173]. They are released from immune and non-immune cells, and are able to positively and negatively regulate the immune response (Table 2 and Figure 3).

With regards to the immune system, a pioneering study described in 2011 by Yang et al. [157], demonstrated that M2 macrophages were able to modulate invasiveness of breast cancer cells through exosome-mediated delivery of miR-223, which directly targets the Mef2c/β- catenin pathway. The breast cancer cells acquired improved capacity for cell migration as a functional consequence of this horizontal genetic transfer. Since the publication of that study, many other miRNAs have been described as critical signaling molecule between tumor and host immune cells. In particular, exosomes-derived miR-940 released by hypoxic epithelial ovarian cancer induce TAM polarization [174]. High levels of miR-203 are present in the serum of colorectal carcinoma (CRC) patients and are associated with later metastasis and overall poor prognosis [175,176]. MiR-203 is shuttled to monocytes where it promotes the expression of M2 markers, thus favoring differentiation to TAM [159].

In the last few years new exosomal “oncomiRs” have been characterized, and it has become more and more evident that cancer-released exosomal miRNAs contribute to both progression of primary tumors and metastases. In particular, miR-21 is expressed and released by both TAMs and solid tumor-derived exosomes [160]. It is shuttled from TAM-derived exosomes to gastric cancer cells, where it suppresses apoptosis and enhances activation of PI3K/AKT signaling pathway by down-regulating PTEN. Moreover, miR-21 is also highly expressed in a wide range of solid tumors [134,177] and secreted in plasma-derived exosomes from patients affected by different cancer types, including pancreatic, ovarian, lung, and colon carcinomas. Enforced expression of miR-21 promotes cellular proliferation, survival, invasion, and migration. Its expression positively correlates with tumor progression and invasiveness [134], while its knockdown decreases tumor cell survival and growth [178]. Interestingly, miR-21 has been described as a key player in a complex regulatory loop between cancer and immune cells, able to support the maintenance of a pro-tumorigenic inflammatory environment. Specifically, the secretion of the pro-inflammatory cytokine IL-6 from immune cells, promotes the invasiveness of tumor cells in an in vitro co-culture model of CRC [179,180]. As result, tumor cells secrete miR-21 and miR-29b into the TME, both of which have been shown to bind to TRLs in macrophages. This leads to the activation of NF-κB signaling pathway and the consequent secretion of pro-metastatic inflammatory cytokines [161]. Exosome-derived miRNAs also participate in cancer promotion by adapting the tumor niche cells. For instance, tumor-secreted miR-9 promotes endothelial cell migration and tumor angiogenesis [181], as does miR-210, an hypoxia-induced miRNA, with increased serum expression levels in breast cancer patients [182]. In this specific context, angiogenesis is enhanced as consequence of miR-210-mediated suppression of the receptor tyrosine kinase ligand ephrin-A3 (Efna3) and increased levels of VEGF [182]. Another example is miR-105, expressed and secreted by metastatic breast cancer cells [169]. miR-105 potently regulates cell migration; by targeting the tight junction protein ZO-1, and thus impairing the integrity of natural cell barriers [169]. More recently, miR-200 family members have been described as able to be internalized by weakly metastatic cells to enhance tumor growth at metastatic lesions [183,184,185]. These findings suggest that horizontal transfer of exosomal miRNAs from cancer cells can facilitate remodeling of tissue architecture to promote cancer progression. Interestingly, several studies reported that cancer cells often secrete onco-suppressor miRNAs via exosomes [186]. Conversely, onco-suppressor miRNAs are less abundant in many solid tumor patients [187,188] suggesting the hypothesis that, tumor suppressor miRNAs might be considered a form of anti-cancer defense mechanism in advanced cancer patients. An interesting example comes from a paper showing that let-7e family members are more highly expressed in exosomes derived from metastatic gastric cancer cells, relative to non-metastatic parental cells [189]. Many other miRNAs with tumor-suppressive functions have been described, including miR-224, miR-921, miR-23b, and miR-15a [190,191,192]. Another intriguing hypothesis supports the idea that in healthy individuals, a certain set of circulating miRNAs, with anti-tumor properties, might be part of a surveillance mechanism, complementary to the canonical cancer immune-surveillance system, exerting continuous inhibition on tumor formation and complementary to the canonical cancer immune-surveillance system [139,193]. This hypothetical action might add a further layer of host anti-tumor defense. However, the relevance of circulating miRNAs in healthy individuals is still unknown; the results presented so far are mainly based on expression profile studies; functional data in animal tumor models are still missing. Extensive studies to identify potential mRNA targets of miRNAs isolated from healthy donors and cancerous patients might provide insights on tumor suppressive or oncogenic mechanisms of circulating miRNAs.

## 7. Immunogenic Cell Death: A Promising Tool for Anti-Cancer Immunotherapy

We pointed out the existence of a dynamic cross-talk between tumors and immune cells that can either leads to progression or regression of the tumor, due to the simultaneous processing of both pro- and anti-growth signals. A major challenge for successful cancer treatment is the understanding of the molecular mechanisms to shift this balance, by favoring the selective killing of cancer cells. The old dogma according to which apoptotic cells do not trigger an immune response has been recently challenged by the emergence of the “immunogenic cell death” (ICD) concept. ICD refers to dying tumor cells, which release endogenous danger molecules, generally called damage-associated molecular patterns (DAMPs), after being exposed to chemotherapeutic agents (reviewed in [194,195]). DAMPs are then recognized by surface receptors expressed on innate immune cells, including pattern-recognition receptors (PRRs), phagocytosis-related receptors, and purinergic receptors, thereby eliciting an anti-tumor immune response. In physiological conditions, most of DAMPs such as calreticulin, adenosine triphosphate (ATP), heat shock protein 90 (HSP90) and high mobility group protein B1 (HMGB1), have predominantly non immunological functions. They are released in response to cellular stress conditions, including production reactive oxygen species (ROS) and endoplasmic reticulum (ER) stress. Interestingly, some of these DAMPs have been demonstrated to be targeted by miRNAs in physiological conditions and few reports also documented a correlation between expression levels of selective miRNAs and induction of tumor cell death by chemotoxic agents. In particular, miR-27a, an oncomiR upregulated in CRC, that directly targets calreticulin has been object of investigation in the study presented by Colangelo et al. in 2016 [196]. The authors demonstrated that upon chemoterapeutic exposure, CRC cells expressing low levels of miR-27 showed upregulated expression of calreticulin and HMGB1, increased release of ATP and enhanced apoptosis [196].

Despite these promising results, several questions remain to be addressed; in particular the molecular mechanisms involved and the impact of miRNA deregulation in the modulation of ICD. Further studies are also needed to define a potential therapeutic use of ICD determinants to trigger effective anti-tumor immune responses.

### miRNAs as Therapeutic Agents in Cancer Treatment

In the last decade, advances in the field of cancer immunology have led to the emergence of immunotherapy as one of the most promising areas of cancer research and treatments [197,198,199,200]. Overall, the goal of cancer immunotherapy is to induce the patient’s immune system to recognize cancer cells as foreign, and destroy them. Various immunotherapies either stimulate specific components of the immune system, or counteract tumor-derived signals that suppress immune responses. Recent advances have been made in the development of T cell-mediated immunotherapies. The most heralded of these are monoclonal antibodies directed at immune checkpoint modulators [199,201], and the clinical realization of chimeric antigen receptor (CAR) T cells [198] (Table 3). On the contrary, few therapies have been aimed at stimulating the response of myeloid cells against cancer [202,203]. In animal models, passive immunization with monoclonal antibodies against tumor antigens can stimulate macrophage-mediated phagocytosis of cancer cells and induce macrophage infiltration into the tumor milieu [201,204].

Compared to other drugs currently on the market for cancer treatment, the intrinsic ability of miRNAs to simultaneously affect gene expression of multiple different pathways makes them an attractive tool for the development of effective drugs. As discussed in the previous sections, miRNAs dysregulation plays a critical role in cancer development, as they are entangled with many of the hallmarks of cancer [205,206]. Therefore, an effective therapeutic strategy might consist in the modulation of specific miRNAs. This can be achieved by specific targeting of cancer-relevant miRNAs at different levels of their biogenesis and activity. Promising results have been obtained in preclinical studies with the use of miRNA antagonists, called antagomiRs, and miRNA mimics to reduce or enhance miRNA activity, respectively (Table 3). AntagomiRs are chemically modified single stranded DNA oligonucleotides complementary to the miRNA sequence, that function as miRNA antagonists and can be employed to repress the activity of oncomiRs, by competing with native tumor-suppressing target transcripts [173]. miRNA mimics are chemically-modified short double-stranded oligonucleotides, that functionally mimic pre-miRNA duplexes; hence they are processed by native cellular pathways to increase expression levels of tumor suppressor miRNAs [155]. To increase their stability and ensure specificity chemical modification have been introduced, including: methoxyethyll-4′-thioRNA (MOE-SRNA), cholesterol-conjugated 2′-*O* methylated miRNA mimic/antagomiRs, and locked nucleic acid (LNA)- modified anti-miRNAs [150].

However, a remaining challenge is the successful delivery of these therapeutic molecules to specific tissue and cells. Indeed, given the pleiotropic role of miRNAs and their ability to function in a cell type-dependent manner, the design of an effective delivery system is critical to guarantee tissue and cell specificity in order to reduce the risk of toxicity and side effects. Different types of biodegradable and biocompatible miRNA carriers have been synthesized as biodegradable and biocompatible, including liposomes, nanoparticles, polymers and viral agents. The versatility of liposomal carriers made them suitable vehicles for co-delivery of miRNAs and small-molecule drugs, which concurrently are able to target the same cancer cell, in an effective synergistic antitumor way. Liposomal carriers were firstly employed for siRNA and small conventional drugs delivery in clinical trials. A liposomal formulation of a mimic of the tumor-suppressive miR-34 was first characterized in animal model of liver cancer [150] and recently reached clinical development. Recently, another miR-34 mimic entered phase I clinical trials for the treatment of advanced hepatocarcinoma [149] (Table 3). Studies have also investigated the use in clinics of viral-based delivery systems [152]. In particular, lentiviral vectors containing antagomiRs against miR-494 have been shown to reduce tumor-infiltrating MDSCs and their protumor activity in an in vivo model of breast cancer [82]. However, the potential risk of therapeutic lentiviral vectors is due to their intrinsic nature to integrate themselves into the human genome. To bypass this risk, adenoviruses and adeno-associated viruses might be more suitable for therapeutic purposes, due to their non-integrative activity. However, limits in large-scale production as well as the immunogenic potential still remain major issues in their effective and safe use in therapy. Therefore, non-viral delivery strategies have received more interest. In particular, cell-derived exosomes containing immune-related miRNAs have the potential to be employed as therapeutic agents. Accordingly, exosome- and immune cell-based delivery represent two interesting potential strategies for miRNA-based cancer immunotherapy. The use of tumor-derived extracellular vesicles to deliver therapeutic miRNAs was recently reported, wherein the authors described the efficient delivery of the tumor suppressive miRNA let-7a to epidermal growth factor receptor (EGFR)-expressing breast cancer cells in vivo. However, the use of exosomes as miRNAs vehicles in cancer therapies is only at the beginning and needs to be further investigated. Possible strategies to improve target selectivity are the modification of the vesicular membrane with ligands or antibodies targeted to the endogenous receptors of tumor or stromal cells. In this context, the combination of miRNA-related immunotherapy with conventional cytotoxic drug agents or targeted therapy would represent a valuable opportunity for effective therapeutic interventions in human malignancies.

## 8. Conclusions

The prominent role of miRNAs as molecular determinants of the innate immune response qualifies them as novel potential therapeutic agents that could critically modulate the fine balance of innate immune cells involved in cancer progression.

## Figures and Tables

**Table 1:** MiRNAs expressed in myeloid cells having an impact on tumorigenesis. MiRNAs having a role in macrophage polarization, tumor invasion and immunosuppression are here listed (↑ = increased; ↓ = decreased).

miRNA	Expression	Targets	Phenotype	
miR-155	↑ M1 macrophages	C/EBPβ, SHIP1, IL13Rα1, SMAD2/3	Reprograms pro-tumoral M2/TAM macrophages to M1 pro-inflammatory macrophages	**Macrophage Polarization**
miR-125b	↑ M1 macrophages	IRF4	↑ responsiveness to IFNγ ↑ tumor killing	
miR-127	↑ in M2 macrophages ↓ by inflammation	DUSP1	↑ M1- and ↓ M2-related genes	
miR-146a	↑ M2 macrophages	NOTCH1, INHBA, PPARγ,	↑ M2 polarization and inflammation ↓ M1 polarization	
miR-223	↓ TAM	IL1β, IL-6	↑ M2 polarization	
let-7c	↑ in M2 macrophages ↓ by inflammation	C/EBPδ, PAK1	↑ M2- and ↓ M1-related genes	
miR-511-3p	↑ TAM	ROCK2	↓ pro-tumoral gene signature of TAMS and ↓ tumor growth	**Tumor invasion**
miR-155	↑ M1 macrophages	SHIP1	↑ anti-tumor immunity. MiR-155 KO myeloid cells induce faster tumor growth	
miR-155	↑ MDSC	SOCS1	Required for tumor growth and the generation of CD4^+^ Treg cells. MiR-155 KO mice are resistant to carcinogenesis	**Immune suppression (MDSC)**
miR-494	↑ MDSC	PTEN	Regulates cell cycle progression; it induces arrest in G2/M and increased inflammation	
miR-20a	↑ MDSC	STAT3	↓ MDSC-dependent suppression of CD4^+^ and CD8^+^ T cell response	
miR-223	↓ MDSC	MEF2C	Suppresses differentiation of tumor induced- CD11bGr1+MDSC	
miR-21	↑ MDSC	SHIP1	↑ proliferation and survival	
miR-690	↑ MDSC	C/EBPα	↑ MDSC expansion and proliferation ↓ terminal differentiation	
miR-17-5p	↑ MDSC	STAT3	↓ MDSC ability to suppress Ag-specific CD4^+^ and CD8^+^ T cell response	

**Table 2:** Exosomal miRNAs functional activities in cancer. Exosomal miRNAs are secreted by macrophages and cancer cells and affect tumor initiation and progression by targeting key factors involved in survival, proliferation, invasion, and angiogenesis. Macrophage- and tumor-derived exosomal miRNAs recently described in the literature are reported here.

miRNA	Donor Cells	Recipient Cells	Phenotype	
miR-21	TAM	gastric cancer cells	↑ cell proliferation ↓ chemosensitivity and apoptosis	**TAM-derived exosomal miRNAs**
miR-223	TAM	breast cancer cells	↑ cell migration capacity and invasiveness	
miR-9	TAM	endothelial cells	↑ cell migration and angiogenesis	
miR-940	epithelial ovarian cancer	TAM	↑ M2 polarization	**Tumor-secreted exosomal miRNAs**
miR-203	colorectal carcinoma	monocytes	↑ M2 polarization	
miR-21	solid tumor cells	macrophages	↑ proliferation, survival, invasion and migration	
miR-29b	solid tumor cells	macrophages	OncomiR: ↑ secretion of pro-metastatic and pro-inflammatory cytokines	
miR-9	tumor cell lines	cancer cells	OncomiR: ↑ endothelial cell migration ↑ tumor angiogenesis	
miR-210	breast cancer cells	adjacent cancer cells	OncomiR: ↑ angiogenesis	
miR-105	breast cancer cells	endothelial cells of distant organs	OncomiR: ↑ metastasis and vascular permeability by targeting the tight junction protein ZO-1	
miR-200	metastatic cancer cells	metastatic cancer cells	Regulates mesenchimal to epithelial transition	
let-7e	gastric cancer cells	cancer cells	Tumor suppressor function (i.e., inhibits metastasis)	
miR-23b	bladder carcinoma cells	cancer cells	Tumor suppressor function (e.g., inhibition angiogenesis, invasion and metastasis)	
miR-224	bladder carcinoma cells	cancer cells	Tumor suppressor functions (e.g., inhibition angiogenesis, invasion and metastasis)	
miR-921	bladder carcinoma cells	cancer cells	Tumor suppressor functions (e.g., inhibition angiogenesis, invasion and metastasis)	
miR-15a	mesenchymal stromal cells	myeloma cells	Tumor suppressor function	

**Table 3:** Immunological drugs in clinical development. List of immunological agents developed for treatment of malignancies. Drugs blocking immune checkpoints, CAR T cell therapies and miRNA-based drugs represent novel promising therapeutics in clinical development.

	Immunological Target	Drug Name	Characteristics	Clinical Phase Testing
**Drugs blocking Immune Checkpoints**	anti-CTLA4	IPILIMUMAB	fully human IgG1	approved, advanced melanoma
		TREMELIMUMAB	fully human IgG2	failed phase III trial melanoma
	anti-PD1	NIVOLUMAB	fully human IgG4	approved, melanoma, squamous NSCLC
		PEMBROLIZUMAB	fully human IgG4	approved, melanoma
		PIDILIZUMAB	humanized IgG1	phase I-II trial
	anti-PD-L1	BMS-936559	fully human IgG4	phase I trial
		MSB0010718C	fully human IgG1	phase I-II trial
		MEDI4736	Fc-modified human IgG1	phase I-III trial
		MPDL3280A	Fc-modified human IgG1	phase I-III trial
**CAR T cells therapy**	anti-CD19	TISAGENLECLEUCEL-T (Kimryah^TM^)	chimeric antigen receptor T cells	approved, Acute lymphatic leukemia
		AXICABTAGENE CILOLEUCEL (Yescarta^TM^)	chimeric antigen receptor T cells	approved, B cell lymphoma
	anti-CD20	NCT01735604	chimeric antigen receptor T cells	phase I trial, progressive malignant lymphoma
	anti-CD30	NCT02259556	chimeric antigen receptor T cells	phase II-III trial, Hodgkin lymphoma
	anti-EGFR	NCT01869166	chimeric antigen receptor T cells	phase II-III trial, Advanced lung cancer
**miRNA-based drugs**	miR-34	MRX34	miRNA mimic	phase I trial, advanced hepatocarcinoma
	miR-122	MIRAVIRSEN	LNA-based antimiRNA	phase II trial, HCV
	miR-29b	MRG-201	miRNA mimic	phase I trial, fibrotic diseases
	miR-155	MRG-106	AntimiRNA	phase I trial, hematological malignances
	miR-10b	-	AntimiRNA	preclinical phase, glioblastoma
	miR-221	-	AntimiRNA	preclinical phase, hepatocarcinoma
